# Identification and genome insights into *Pyrenochaeta nobilis*, a novel endophytic fungus isolated from *Astragalus membranaceus* with gray mold-control activity

**DOI:** 10.3389/fpls.2025.1610287

**Published:** 2025-10-16

**Authors:** Fan Yang, Shuang Wang, Xifeng Jiang, Hongrui Du, Yu Liu, Yuanyuan Zhou, Chunlai Liu

**Affiliations:** ^1^ Department of Biological Control, Plant Protection Institute of Heilongjiang Academy of Agricultural Sciences, Harbin, Heilongjiang, China; ^2^ Department of Biological Observation, Harbin Crop Pest Scientific Observing and Experimental Station of the Ministry of Agriculture, Harbin, Heilongjiang, China; ^3^ Department of Plant Conservation, Heilongjiang BaYi Agricultural University, Daqing, China

**Keywords:** biocontrol agent, *Botrytis cinerea*, fungal endophyte, inhibitory effect, genome assembly, *Pyrenochaeta nobilis*

## Abstract

**Introduction:**

Gray mold, caused by the necrotrophic fungus *Botrytis cinerea*, is a significant threat to agricultural production, especially under low temperature and high humidity conditions. This disease can cause substantial yield losses in various crops, including tomatoes. To address this issue, the search for novel biocontrol agents has become a priority. In this study, we explored the potential of endophytic fungi isolated from wild medicinal plants in the southern foothills of the Daxing’an Mountains in China as biocontrol resources against *B. cinerea*.

**Methods:**

Endophytic fungi were isolated from the roots of *Astragalus membranaceus*, a wild medicinal plant native to the study area. Among the isolates, *Pyrenochaeta nobilis* strain SFJ12-R-5 (CGMCC No.17766) was selected for its significant antagonistic activity against *B. cinerea*. The inhibitory effects of *P. nobilis* on *B. cinerea* were evaluated through in vitro assays, including mycelial growth inhibition tests and lesion inhibition tests on tomato leaves and fruits. Additionally, the genome of *P. nobilis* SFJ12-R-5 was sequenced using a combination of next-generation and third-generation sequencing techniques, followed by systematic annotation and identification of key gene families, such as carbohydrate-active enzymes (CAZymes) and phage-related (Phi) genes.

**Results:**

*P. nobilis* strain SFJ12-R-5 exhibited strong inhibitory effects on *B. cinerea*, with a mycelial growth inhibition rate of 66.67 ± 3.15% and a large inhibition zone of 20.83 ± 3.78 mm. The fresh fermentation filtrate of *P. nobilis*, even at a 10-fold dilution, completely inhibited the growth of pathogenic hyphae. In vitro tests on tomato leaves and fruits showed lesion inhibition rates of 87.21% and 100%, respectively. Furthermore, plants co-treated with *B. cinerea* and the *P. nobilis* filtrate had a significantly lower gray mold disease severity (28.57%) compared to those inoculated solely with *B. cinerea* (75.34%), indicating a disease reduction rate of 62.08%. The genome of *P. nobilis* SFJ12-R-5 was successfully assembled and annotated, revealing the presence of CAZymes and Phi genes that may contribute to its biocontrol potential.

**Discussion:**

Our findings provide the first evidence that *P. nobilis* could serve as a promising natural antagonist against *B. cinerea*, particularly in integrated disease management systems for tomato production in greenhouses. The high-quality genome sequence and the identification of key gene families lay a solid foundation for future research on the molecular mechanisms underlying the inhibitory activity of Pyrenochaeta spp. against *B. cinerea*. Further studies are needed to explore the practical application of *P. nobilis* in agricultural settings and to elucidate its mode of action at the molecular level.

## Introduction

Necrotrophic fungus are pathogenic microorganisms that kill host cells to feed on the dead tissues by secreting toxins or degrading enzymes. The necrotrophic fungus *Botrytis cinerea*, which is a type of fungal pathogen that can first cause the death of host plant cells and then feed on the dead tissues, causes gray mold disease in tomatoes. Currently, it has infected a great variety of important plant species globally, including vegetables (Alkilayh et al., 2024; Abbasi et al., 2025; Motallebi, 2025), fruits (Dai et al., 2024; Lagrèze et al., 2025; Liang et al., 2025), and flowers (Ha et al., 2025; Hamedan et al., 2025) planted in glasshouses. Gray mold is a devastating disease in many tomato-growing regions, leading to significant economic losses and reduced production (Borges et al., 2015; Boukaew et al., 2017; Ahmadu et al., 2021; Li et al., 2023b). Pathogen infection can occur during harvesting, handling, storage and even after consumer purchase (Romanazzi et al., 2016). Severe infections result in extensive defoliation, flower drop reduced fruit quality and stem cankers, ultimately leading to plant death (Dik et al., 1999). In extreme cases, gray mold can reduce farm yields by 40–50% or even cause complete crop failure (Thole et al., 2020).

*B. cinerea* is a highly adaptable pathogen, characterized by abundant spore production, rapid genetic variation, a short life cycle, and strong transmission ability. It has also developed significant resistance to multiple fungicides (Xue et al., 2013; Ge et al., 2015; Lian et al., 2017). Currently, resistant tomato cultivars are limited and often exhibited undesirable horticultural traits (Bestfleisch et al., 2015). To date, gray mold control has relied heavily on chemical fungicides due to the lack of resistant germplasm and commercial varieties (Sarven et al., 2020; Wang et al., 2020b). However, fungicide resistance in *B. cinerea* is rapidly increasing to overuse, diminishing the effectiveness of conventional chemical control strategies (Liu et al., 2016). Moreover, extensive fungicides application contributes to environmental pollution, human health hazards, and further resistance evolution in pathogens. Therefore, there is an urgent need for eco-friendly and sustainable disease management strategies, including the use of microbial biocontrol and their metabolites (Bolívar-Anillo et al., 2019; Hassan et al., 2021).

The International Biocontrol Manufacturers Association (IBMA) defines biocontrol agents naturally derived products that suppress crop pests or pathogens by inhibiting their growth or reproduction. These agents include macroorganisms, microorganisms, chemical mediators, and natural substances (Lecomte et al., 2016). Among them, bacteria and fungi are shown great potential as biocontrol agents, with an increasing number of antagonistic strains been utilized to enhance plant growth and disease resistance (Köhl et al., 2020; Rojas et al., 2020; Li et al., 2023a; Feng et al., 2024).

Compared with biocontrol bacteria, biocontrol fungi exhibit distinct advantages in biological control: they possess a broader host range, exert inhibitory effects through the synergistic action of multiple biocontrol mechanisms, and demonstrate stronger environmental resilience (Song et al., 2025). Fungal endophytes are naturally occurring symbiotic microorganisms that colonize plant tissues without causing visible damage (Bolívar-Anillo et al., 2019). These microorganisms can enhance plant defenses against insects and disease while also improving tolerance to environmental stresses through the production of bioactive metabolites. Medicinal plants are rich source of biologically active compounds, some of which may be derived from their endophytic fungi (Kumar and Kaushik, 2013; Alvin et al., 2014). However, the full potential of these fungi remains largely unexplored (Deshmukh et al., 2014).

The construction of high-quality genomes serves as the foundation for studying species functional mechanisms. Although short-read sequencing currently boasts high single-base accuracy, its short read length results in poor assembly contiguity. In contrast, while long-read sequencing alone can achieve better assembly contiguity, the lower single-base accuracy of its sequencing data leads to frequent single-base errors in the assembly results (Warburton and Sebra, 2023; Li and Durbin, 2024). Currently, numerous genomics studies have integrated these two types of data to leverage their respective strengths, high accuracy from short reads and excellent contiguity from long-read sequencing, thereby ensuring the generation of high-quality genome assemblies suitable for subsequent functional analyses (Shi et al., 2024; Yu et al., 2024; Zhu et al., 2025).

In this study, we hypothesize that new species with inhibitory activity against *B. cinerea* can be screened from the habitats of certain medicinal plants; more specifically, we propose that natural endophytes with high inhibitory activity against the gray mold pathogen *B. cinerea* exist in such environments. Medicinal plants such as *Astragalus membranaceus* can biosynthesize bioactive compounds including flavonoids, isoflavonoids, and triterpenoid saponins (Li et al., 2024). These substances may act as “intermediate mediators” to recruit growth-promoting microorganisms, thereby enhancing resistance against pathogens (Zhou et al., 2022). There are also previous reports indicating that several endophytic species have been successfully isolated and screened from the leaves of this plant (Kim et al., 2017). Guided by this hypothesis, we conducted a screening experiment using *A. membranaceus*, a widely studied medicinal plant, as the target host. Ultimately, we successfully isolated a novel biocontrol fungal endophyte, *Pyrenochaeta nobilis*, which exhibits strong inhibitory activity against *B. cinerea*. Through co-culture assays, we confirmed its antagonistic effect on *B. cinerea* and comprehensively evaluated its protective efficacy on tomato leaves, fruits, and whole plants. Furthermore, using second- and third-generation sequencing technologies, a high-quality genome was constructed and the phylogenetic relationships of this species was analyzed.

## Materials and methods

### Isolation of fungal endophytes

Plant tissues were surface-sterilized using the procedure described by Eze et al. (2019) with minor modifications (Eze et al., 2019). The root, stem, leaf and flower segments were rinsed under running tap water. After air-drying, the cleaned stems and roots were cut into small pieces, and then all the tissues were surface sterilized by immersion in 70% ethanol (Merda, Beijing, China) for 1 min, followed by 2% sodium hypochlorite solution (Merda, Beijing, China) for 2 mins, and subsequent washing three times in sterile distilled water. The surface-sterilized samples were cut into smaller pieces using a sterile blade and placed on sterile potato dextrose agar (PDA) (Franklin Lakes, NJ, USA) at 25°C. The hyphal tips of endophytic fungi growing out from the plant tissues were transferred to PDA plates supplemented with streptomycin (400 μg/mL) to inhibit bacterial growth until mycelia or colonies appeared around the segments. The efficiency of the surface sterilization procedure was checked for each sterilized plant segment using the imprint method. Additionally, to detect the presence of surface associated fungi, non-surface-sterilized plant samples were cultured under the same conditions as negative controls. All isolated endophytic fungi were screened for antagonistic strains using the plate confrontation method (Li et al., 2015).

### Isolates and cultural conditions

The endophytic fungi *P. nobilis* SFJ-12-R-5 (NCBI accession: MT568589) was isolated from the root of *Astragalus membranaceus* in Xinsheng Village, Sanchahe Town, Arong Banner, Hulunbuir City, Inner Mongolia Autonomous Region, located in the eastern region of the Greater Hinggan Mountains of China (122°2′30″-124°5′40″E, 47°56′54″-49°19′35″N). The strain was preserved in the China General Microbiological Culture Collection Center (CGMCC No.17766).

The SFJ-12-R-5 isolate was used as an antagonistic strain. The first-generation purified strain stored on a slant was activated on PDA at 25°C in the dark for 14 days. The pathogen strain, *Botrytis cinerea*, was incubated on PDA at 23°C with 70% relative humidity in a dark incubator (Bilon, Shanghai, China) for five days. To prepare the fermentation broth, SFJ-12-R-5 was cultured in a 500 mL Erlenmeyer flask containing 150 mL potato dextrose broth (PDB) with three 7 mm mycelial disks at 25°C and 180 rpm on a rotary shaker (Bilon, Shanghai, China) for 14 days. The culture broth was then centrifuged at 8,000 rpm for 15 min to remove the mycelium, and the supernatant was collected. The supernatant was further filtered with a 0.22 µm membrane filter (Millipore Sigma, USA) to obtain a sterile filtrate for subsequent use. 25% Pyrisoxazole emulsifiable concentrate (EC) (Guoguang, Chengdu, China) was used as the chemical control.

### The inhibitory effect of *P. nobilis* SFJ-12-R-5

The dual-culture confrontation assay was used to evaluate the inhibitory effect of strain SFJ-12-R-5 against *B. cinerea*. A 7 mm diameter mycelial disk of SFJ-12-R-5 was placed on a fresh PDA plate, 1 cm from the edge of the petri dish, while a 7 mm mycelial disk of *B. cinerea* was placed on the opposite side. For the control group, only a single *B. cinerea* mycelial disk was placed on a PDA petri dish. All plates were incubated at 23°C in the dark until the growth of *B. cinerea* reached the edge of the control petri dish. Each treatment was performed in triplicate. The inhibition of mycelial growth was calculated using the following formula:


$$\;\text{IGM}(\%)=[\frac{{\text{d}}_{\text{c}}-{\text{d}}_{\text{t}}}{{\text{d}}_{\text{c}}}]\times 100\%$$

where IGM denotes the inhibition ratio of *B. cinerea* mycelia growth, while the *d_c_* and *d_t_* indicate the growth diameters of *B. cinerea* in the control and SFJ-12-R-5 treatment groups, respectively (Park et al., 2015).

### The effect of *P. nobilis* SFJ-12-R-5 filtrate against the mycelia growth of *B. cinerea*

The Oxford cup assay was used to evaluate the inhibitory effect of SFJ-12-R-5 culture filtrate on *B. cinerea* mycelial growth. A 100 μL spore solution of *B. cinerea* was evenly spread onto a PDA petri dish, and three Oxford cups were placed equidistantly on the plate. After approximately 3 hours of incubation, 250 μL of SFJ-12-R-5 culture filtrate was added to each cup. For the control group, only the 100 μL *B. cinerea* spore solution was spread onto the PDA plate without filtrate treatment. All plates were then incubated at 23 °C in the dark until the growth of *B. cinerea* reached the edge of the petri dish. Each treatment was performed in triplicate, and the diameters of the inhibition zones were measured.

A second filtrate dilution assay was conducted to evaluate the effect of different filtrate concentrations. Various volumes of SFJ-12-R-5 filtrate were mixed with PDA medium to create plates containing serial dilutions (1:10, 1:50, 1:100, 1:500, and 1:1000). Sterile PDB mixed with PDA medium served as the control. A 7 mm mycelial disk of *B. cinerea* was placed in the center of each petri dish, and all plates were incubated at 23°C in the dark until the growth of *B. cinerea* reached the edge of the control petri dish. Each treatment was conducted in triplicate, and the inhibition ratio of *B. cinerea* mycelia growth was calculated (Park et al., 2015).

### The biocontrol efficacy of SFJ-12-R-5 filtrate against *B. cinerea* on detached cherry tomato leaves

Healthy cherry tomato leaves were disinfected by soaking in a 1% NaCl solution for 2 minutes, followed by thorough rinsing with sterilized water. After air-drying, ten leaves were wrapped at the petiole with moistened sterile cotton and placed on moistened sterile filter paper in a germination box. A 5 mm mycelial disk of *B. cinerea* was inoculated onto each leaf, and 10 mL of SFJ-12-R-5 culture filtrate was sprayed per box. For the pathogen control, PDB medium was used instead of SFJ-12-R-5 filtrate. 10 mL of 120 μg/mL 25% pyrisoxazole EC was sprayed in the chemical control box. For the blank control, leaves not inoculated with *B. cinerea* were sprayed only with sterile water. All boxes were cultured at 23°Cunder a 12-hour light/12-hour dark (L:D=12:12) cycle. Six days post-incubation, lesion diameters were measured. Each treatment was performed in triplicate, with ten leaves per replicate, and the experiment was repeated three times.

### The biocontrol efficacy of SFJ-12-R-5 filtrate against *B. cinerea* on detached cherry tomato fruits

Healthy tomato fruits of similar sizes were disinfected by soaking in a 1% NaClO solution for 2 minutes, followed by thorough rinsing with sterilized water. After air-drying, fifteen fruits were then placed on moistened sterile filter paper in a germination box. Each disinfected tomato fruits were wounded (2 mm depth, 2 mm in diameter) using a sterile nail. The treated fruits were then air-dried on a clean bench, and 2 μL of a *B. cinerea* conidial suspension (1×10^6^ conidia/mL) was pipetted into each wound (Hua et al., 2019). Approximately one-hour post-inoculation, 6 μL of SFJ-12-R-5 culture filtrate and 6 μL of 120 μg/mL 25% pyrisoxazole EC was respectively dropped into the fruit wounds of the corresponding treatment boxes. For the blank control, fruits not inoculated with *B. cinerea* conidia were treated only with sterile water. All boxes were cultured at 23 °C under a L:D=12:12 cycle. Six days post-incubation, lesion diameters were measured. Each treatment was performed in triplicate, with 15 fruits per replicate, and the experiment was repeated three times.

### The biocontrol efficacy of SFJ-12-R-5 filtrate against *B. cinerea* on cherry tomato plant

Tomato seeds were sown in soil and transplanted into individual pots after three weeks. The plants were grown in a greenhouse for six weeks. Afterward, each plant of similar size was inoculated with 4 mL of a *B. cinerea* conidial suspension (1×10^6^ conidia/mL). Fifteen minutes post-inoculation, each plant was sprayed with 4 mL of SFJ-12-R-5 culture filtrate until runoff. Plants inoculated only with *B. cinerea* conidial suspension served as the pathogen control, while those sprayed with sterile distilled water were treated as the blank control. 4 mL of 120 μg/mL 25% pyrisoxazole EC was sprayed on each plant as a chemical control. The experimental plants were arranged in a complete randomized block design with two replicates of three plants per treatment. Six days post-inoculation with *B. cinerea* conidia, the gray mold incidence was recorded. Gray mold severity on tomato plants was measured via a 0–4 disease-rating scale (Lee et al., 2006).

### DNA extraction and genome sequencing

The genome was sequenced by Majorbio Biotech Co., Ltd (Shanghai, China). Briefly, genomic DNA was extracted using the CTAB method. DNA concentration and integrity were assessed using a Qubit 3.0 fluorometer (Thermo Fisher Scientific, United States) and Nanodrop 2000 (Thermo Fisher Scientific, United States), respectively. A library with insert sizes of approximately 350 bp was constructed following Illumina’s second-generation sequencing library preparation standards. The library was then sequenced on the Illumina NovaSeq 6000 platform (Illumina Inc., San Diego, CA, United States) with a PE150 layout. Additionally, a 20-kb SMRT Bell library was constructed using the DNA Template Prep Kit 1.0, and sequenced on the PacBio Sequel system (Pacific Biosciences, United States).

### Genome assembly and annotation

Following sequencing, data quality control was performed using FASTP (v.0.20.0) (Chen et al., 2018) with default parameters. This process involved removing low-quality reads, adapter sequences, and reads containing more than 10% ambiguous nucleotides (N). GenomeScope (Vurture et al., 2017) was then employed to assess the genomic characteristics based on the short reads obtained from the sequencing. Canu (v1.7) (Koren et al., 2017) was used to assemble the genome. The genome was further polished using Pilon (v1.24) (Walker et al., 2014) with short reads over three rounds of refinement. To evaluate genome completeness, BUSCO (v5.8.1) (Simão et al., 2015) was applied with the fungi_odb10 dataset. GC-Depth values were calculated and visualized using a custom R script. Repeat elements within the genome were annotated using RepeatMasker (v4.0.7) (Tarailo-Graovac and Chen, 2009). Gene prediction was performed with MAKER2 (v2.31.9) (Holt and Yandell, 2011). Ribosomal RNA (rRNA) and transfer RNA (tRNA) components were predicted using Barrnap (v0.4.2) and tRNAscan-SE (v1.3.1) (Lowe and Eddy, 1997), respectively.

To annotate the functions of the predicted coding genes, amino acid sequences were aligned to NR (Latest), Swiss-Prot (v20170410) (Gasteiger et al., 2001), Pfam (v31.0) (Finn et al., 2006) and KEGG (Latest) using BLAST (v2.3.0) (Camacho et al., 2009). The gene ontologies (GO) were annotated using Blast2GO (V2.5) (Conesa et al., 2005). Carbohydrate active enzymes (CAZymes) were annotated using the CAZy (v6) (Cantarel et al., 2009) database. Genes related to host interactions were annotated with the PHI-base (Urban et al., 2020) (v4.4) database, and virulence factors were identified through the DFVF (v1.0) (Lu et al., 2012) database. Secreted proteins were identified using SignalP (v6) (Teufel et al., 2022), and transporters were annotated using the TCDB (Saier et al., 2021) database. The secondary metabolite related genes were predicted with antiSMASH (v8.0.2) (Blin et al., 2019). Additionally, transmembrane proteins were also considered during the analysis. The genome characteristics were illustrated using Circos (v0.69-6) (Krzywinski et al., 2009).

### Phylogenetic analysis

To elucidate the evolutionary relationships of *P. nobilis*, ten related species were selected for comparative analysis. Orthofinder (v2.27) (Emms and Kelly, 2019) was used to identify orthologous genes among the 11 species. The Upset tool (Lex and Gehlenborg, 2014) was employed to visualize the shared and unique gene families across all species. Subsequently, orthologous genes were aligned using Muscle for multiple sequence alignment (Edgar, 2004), and the alignment results were concatenated to generate the super-gene sequence. The phylogenetic tree was then constructed using MEGA (v11) (Tamura et al., 2021) with the Tamura-Nei model and 1000 bootstrap replicates. Finally, the tree was visualized using iTOL (https://itol.embl.de/) (Letunic and Bork, 2021).

### Statistical analysis

Data were analyzed for statistical significance using one-way analysis of variance (ANOVA). Mean comparisons were performed using the least significant difference (LSD) test at P ≤ 0.05. Statistical analyses were conducted using SPSS software (version 19.0, SPSS Inc., Chicago, USA).

## Results

### The inhibitory effect of *P. nobilis* SFJ-12-R-5 on the mycelial growth of *B. cinerea*

**Figure 1:**
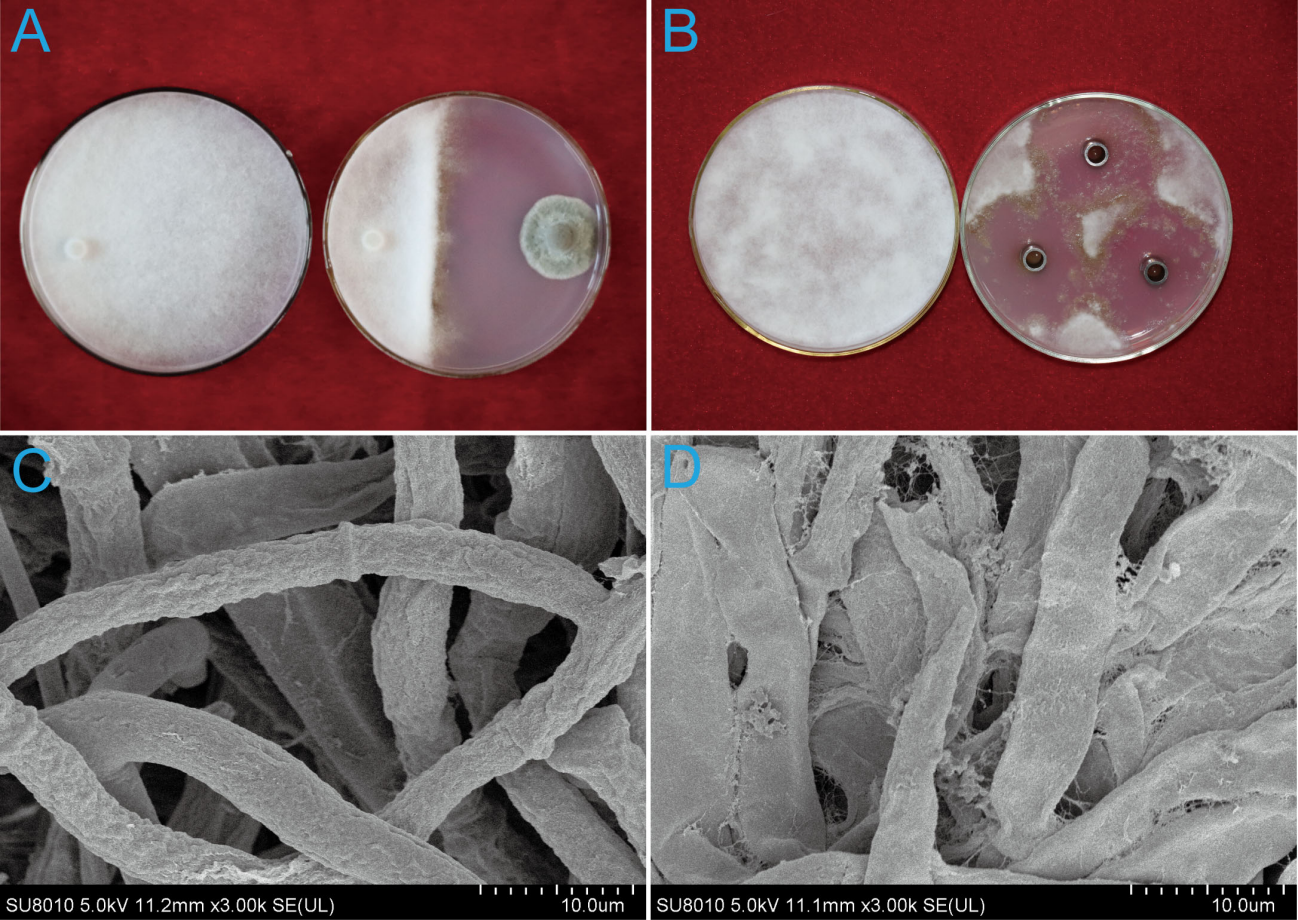
Effects of strain SFJ-12-R-5 and its metabolites on the mycelial growth of *Botrytis cinerea*. **(A)** Single-strain culture of SFJ-12-R-5 and *B cinerea*. **(B)** Inhibitory effect of SFJ-12-R-5 metabolites on *B cinerea*. **(C)** Untreated control showing normal mycelial growth. **(D)** Morphological changes in *B. cinerea* hyphae after treatment with SFJ-12-R-5 metabolites.

*P. nobilis* SFJ-12-R-5 produced a significant inhibition zone in front of the *B. cinerea* colony (**Figure 1A**), reducing mycelial growth by 66.67 ± 3.15%. Furthermore, the hyphae at the edge of the *B. cinerea* colony appeared sparse and loosely arranged, suggesting the presence of fungistatic secondary metabolites produced by *P. nobilis*.

### The effect of *P. nobilis* SFJ-12-R-5 filtrate on the mycelia growth of *B. cinerea*

No hyphae were observed around the Oxford cups. Colonies grown on PDA supplemented with 250 μL of culture filtrate in Oxford cups were significantly inhibited compared to the control treatment. The inhibition zone was notable, with a diameter of 20.83 ± 3.78 mm (**Figure 1B**). Control hyphae were uniformly distributed, intact and robust, and (**Figure 1C**). In contrast, the treated hyphae exhibited abnormalities, including excessive twisting, short branches, and cell wall lysis (**Figure 1D**).

**Figure 2:**
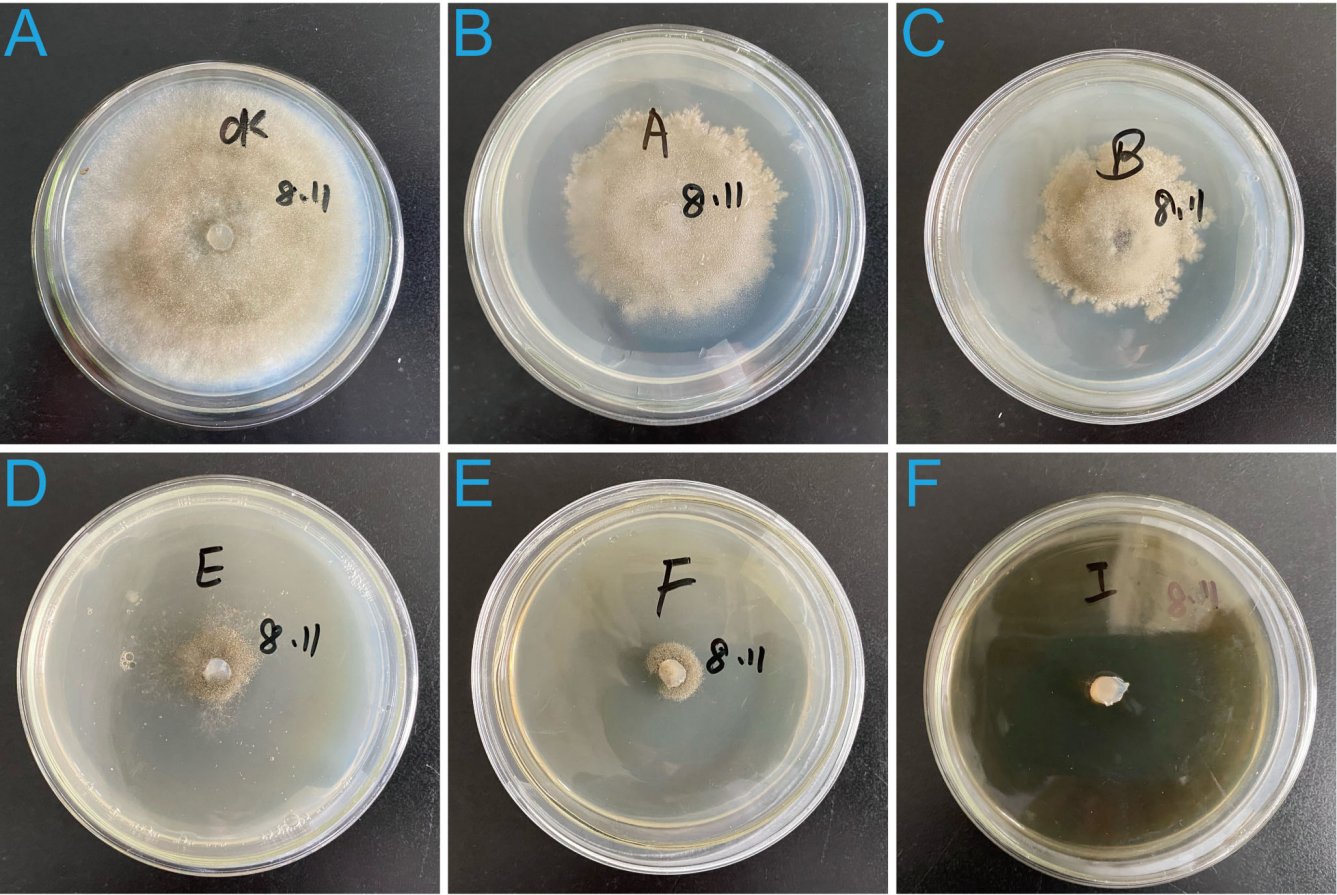
Inhibitory effects of strain 12-R-5 fermentation filtrate on the mycelial growth of *B cinerea* at different dilutions levels. Filtrate dilutions: 1000-fold **(B)**, 500-fold **(C)**, 100-fold **(D)**, 50-fold **(E)**, and 10-fold **(F)**. **(A)** Untreated control showing normal mycelial growth.

The filtrate inhibited mycelial growth in a concentration-dependent manner, and with significant differences observed among different filtrate dilutions (**Figures 2A–F**). The inhibition rates of *B. cinerea* mycelial growth were 100 ± 0%, 89.14 ± 1.35%, 82.34 ± 0.35%, 45.24 ± 1.46%, and 35.65 ± 1.16% when treated with filtrate diluted 10, 50, 100, 500, and 1000 times, respectively, all significantly (*p* < 0.001) higher than the control group (0%).

### The culture filtrate of *P. nobilis* SFJ-12-R-5 reduced gray mold severity on detached leaves, fruits and whole tomato plants

**Figure 3:**
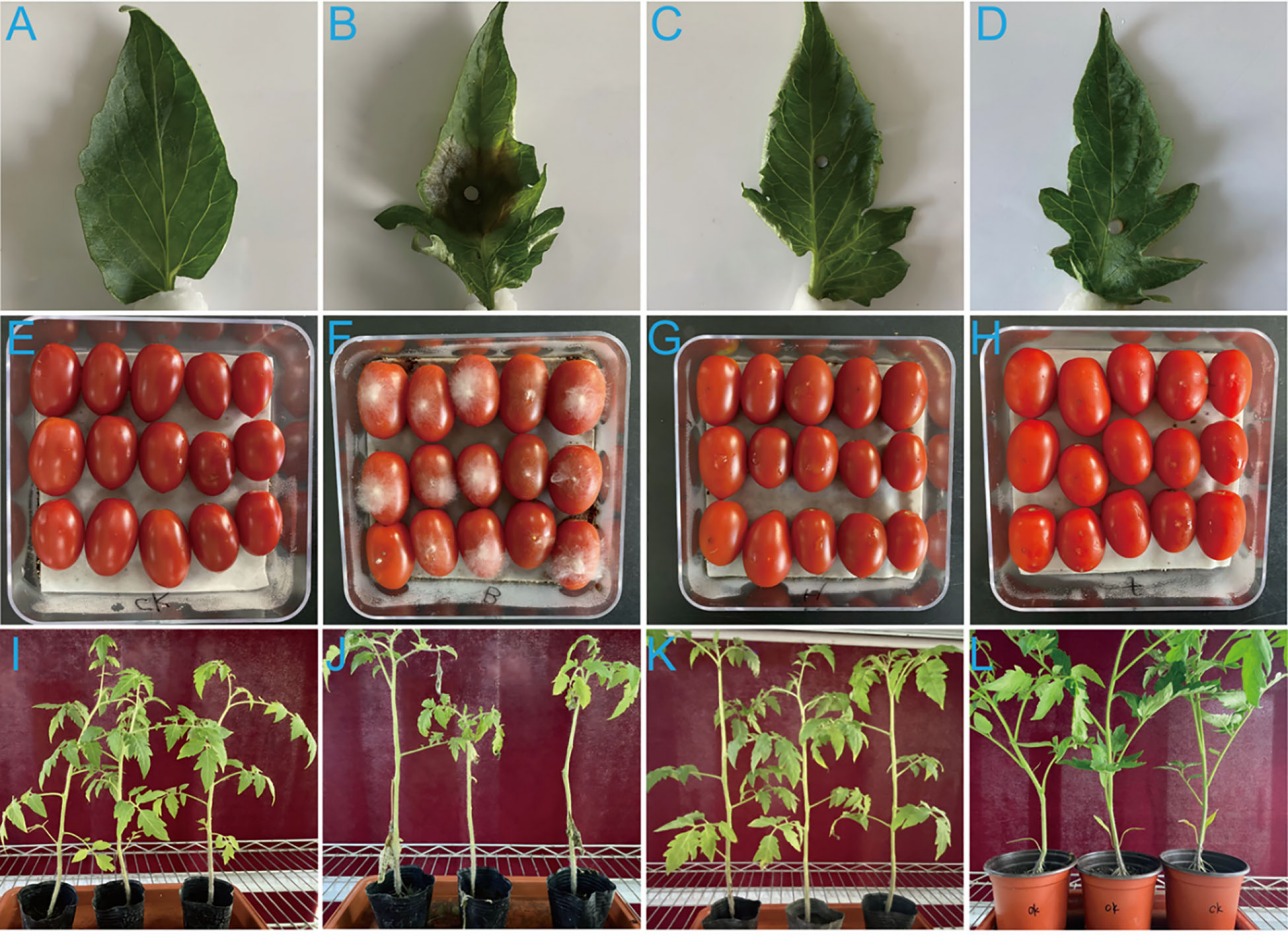
Biocontrol efficacy of strain SFJ-12-R-5 fermentation filtrate against *Botrytis cinerea* on detached tomato leaves, fruits, and whole plants. **(A, E, I)** Untreated controls; **(B, F, J)** Pathogen controls without filtrate treatment; **(C, G, K)** Samples treated with SFJ-12-R-5 fermentation filtrate; **(D, H, L)** Samples treated with 25% pyrifenox emulsifiable concentrate (EC).

Leaves inoculated only with *B. cinerea* exhibited typical gray mold lesions, where leaves treated with biocontrol agent treatment and chemical agent treatment groups showed significant reduced lesions (**Figures 3A–D**). The lesion area in the pathogen control group was 6.34 cm^2^, while in the filtrate-treated group, it was only 0.81 cm^2^, corresponding to an 87.21% inhibitory effect (*p*<0.01). The area of leaf lesions in the chemical agent treatment group was 0.64 cm^2^, and the inhibitory effect was 89.90% (*p*<0.01). The biocontrol potential of *P. nobilis* filtrate was also tested on cherry tomato fruits. Fruits treated only with B. cinerea exhibited a higher incidence of gray mold compared to those treated with both *B. cinerea* and filtrate. The lesion area on pathogen control fruits was 4.32 cm^2^, while no lesions were observed on fruits treated with biocontrol agent and chemical agent treatment groups. The inhibition rate of both treatment groups was 100% (**Figures 3E–H**). When comparing treatment groups, plants treated only with *B. cinerea* showed a gray mold severity of 75.34%, notably higher than the 28.57% observed in plants treated with both *B. cinerea* and the filtrate. This combined treatment achieved a 62.08% reduction in gray mold severity (**Figures 3I–L**). These results suggest that *P. nobilis* effectively reduced the lesions caused by *B. cinerea* on both leaves and fruits.

### The genome of *P. nobilis* SFJ-12-R-5

The genome was sequenced using a combination of NGS and Pacbio SMRT sequencing technologies. For Illumina sequencing, 13.98 million paired-end (PE) 150 reads were generated, corresponding to 4.25 Gb of data (**Supplementary Table S1**). Pacbio sequencing yielded 5.12 Gb of data, with a total of 512.99 thousand reads and an average read length of 9,980 bp (**Supplementary Table S2**).

**Figure 4:**
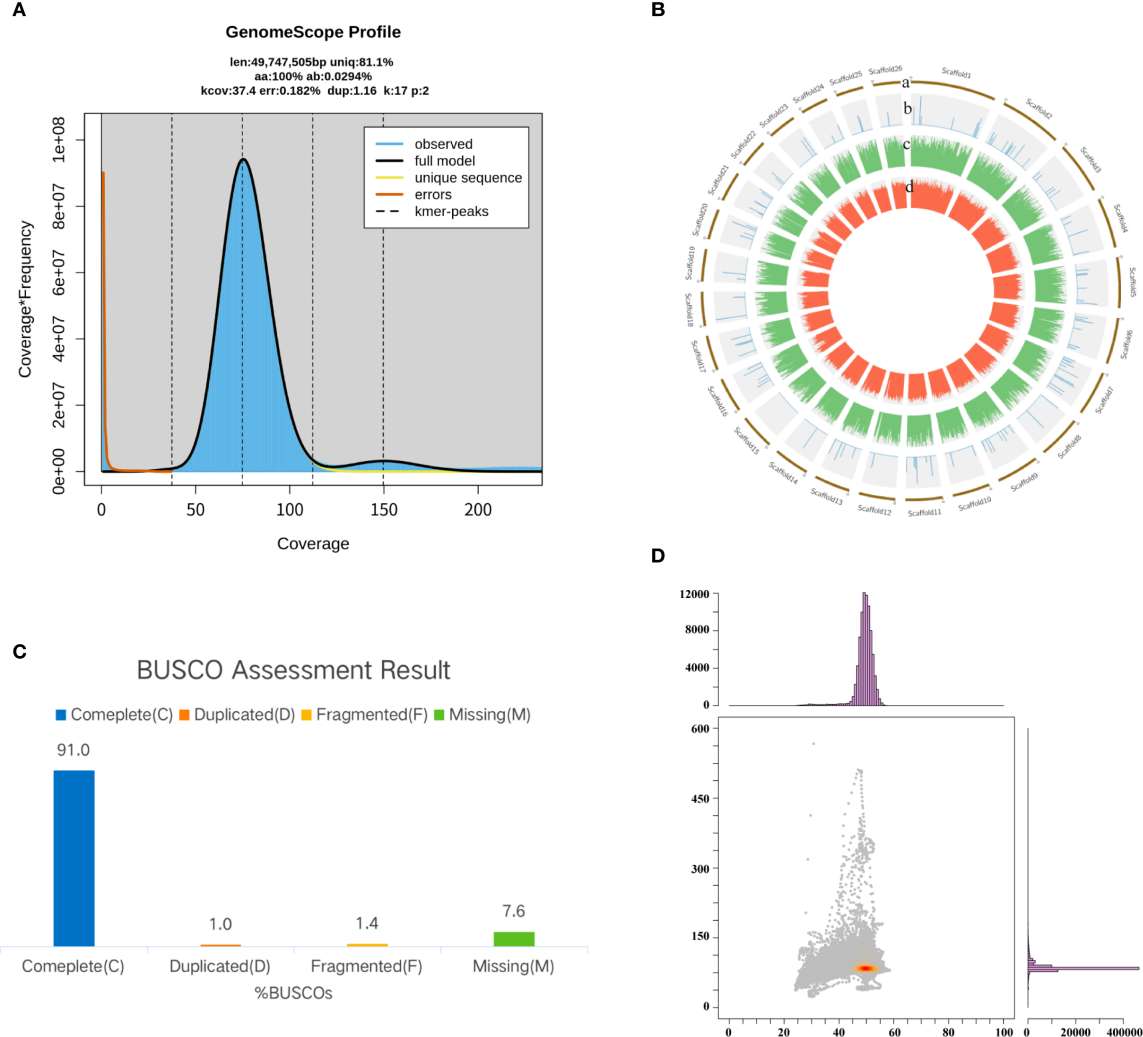
Genome assembly and quality assessment of *P. nobilis* SFJ-12-R-5. **(A)** Genome characteristics survey using GenomeScope. **(B)** A Circos plot showing the genome components. **(C)** Genome completeness assessment using BUSCO. **(D)** A GC-depth plot showing the quality of assembled sequences.

Based on the analysis of *k*-mer distribution, the estimated genome size was 49.75 Mb. The genome displayed a low heterozygosity level of 0.03% and a repeat sequence content of 18.90% (**Figure 4A**). After assembly, the final genome size was 42.59 Mb, comprising 115 contigs with a GC content of 49.16% (**Figure 4B**; **Table 1**). The quality of the assembly was reflected in a contig N50 value of 435.95 kb and an average contig length of 370.25 kb. Genome completeness, assessed using the BUSCO database, revealed that the assembly achieved 91% completeness (**Figure 4C**). Additionally, the GC-depth profile showed an even distribution of sequencing depth across varying GC percentages, with no evidence of contamination from other species (**Figure 4D**).

**Table 1:** Genome features of *P. nobilis* SFJ-12-R-5 genome.

Indexes of genome sequence	Values
Assembly genome length (Mb)	42.58
Number of contigs	115
GC content (%)	49.16
Average contig length (Kb)	370.25
Max contig length (Kb)	1,603.87
Contig N50 (Kb)	435.95
Contig N90 (Kb)	206.76
Annotated genes	14,578
Average gene length (bp)	1,855
Gene density (number/Kb)	0.34
Gene length/Genome length (%)	63.51

### Genome annotation of *P. nobilis* SFJ-12-R-5

The proportion of repetitive sequences in the genome was exceptionally low, constituting only 10.86%. A total of 14,578 protein-coding genes were identified in the genome (**Supplementary Table S3**), of which 11,519 (79.02%) were successfully annotated using existing databases (**Table 2**; **Supplementary Table S3**). The total length of all protein-coding genes was 27.04 Mb, accounting for 63.49% of the total genome length. On average, each protein-coding gene was 1,854.87 bp long, with an average of 5 exons per gene. Additionally, 73 tRNAs and 25 rRNAs were identified in the genome.

**Table 2:** Gene function annotation of *P. nobilis* SFJ-12-R-5 genome.

Databases	Number	Percentage
KEGG	1,940	13.31%
GO	6,645	45.58%
Swiss-Prot	6,723	46.12%
Pfam	7,587	52.04%
COG	10,076	69.12%
NR	11,517	79.00%
Total annotated	11,519	79.02%
Total genes	14,578	100.00%

**Figure 5:**
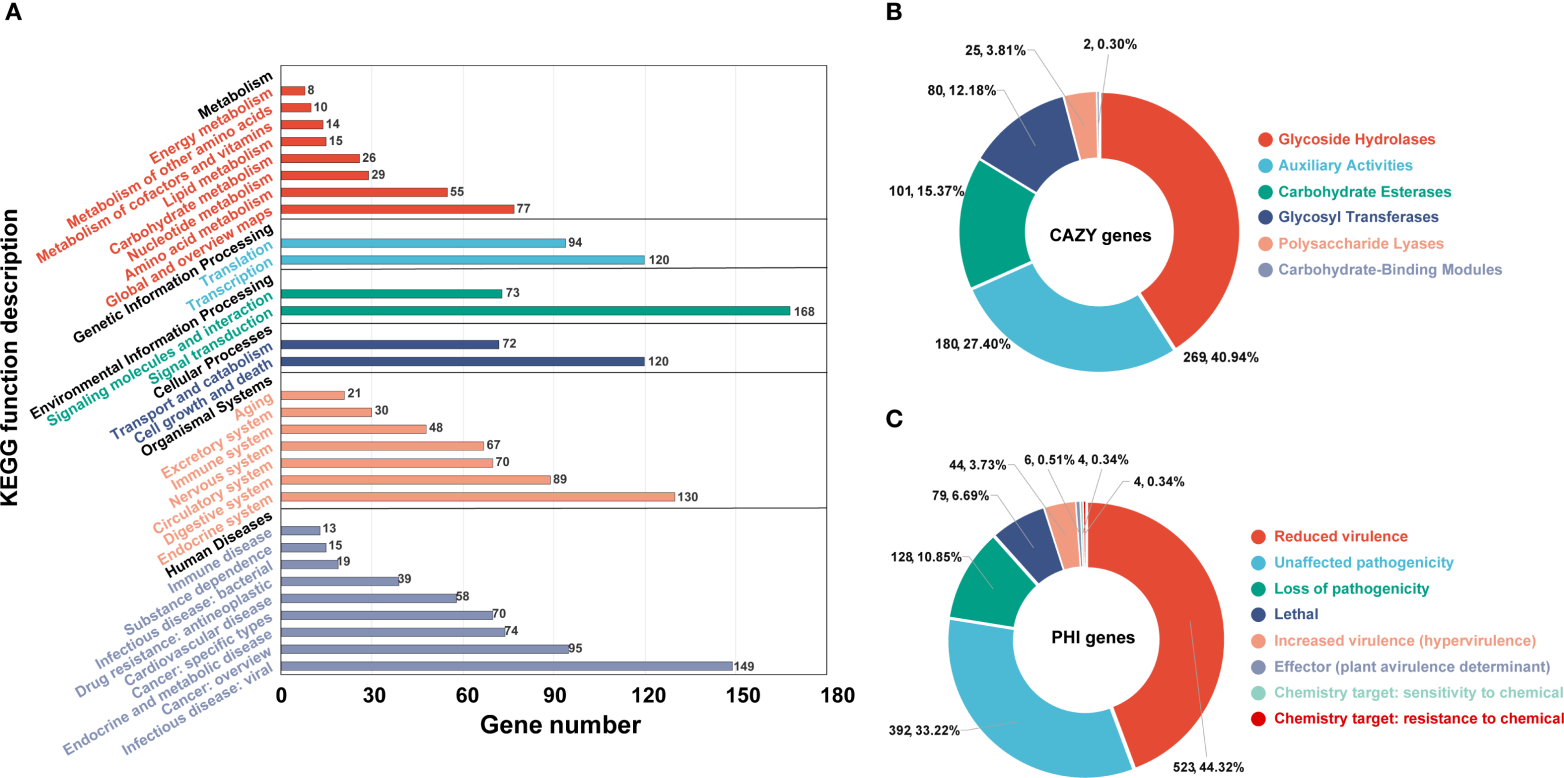
Gene function annotation of *Pinna nobilis* SFJ-12-R-5 using different databases. **(A)** Statistics of the Class II functions in the KEGG database. **(B, C)** show the annotation function statistics of the CAZy and Phi databases respectively.

Further annotation of the predicted coding genes was performed using multiple databases, yielding the following results. KEGG annotation revealed that the genes were primarily distributed across 30 subcategories within 6 major categories (**Figure 5A**). The top three subcategories, based on gene count, were signal transduction (168 genes), infectious diseases: viral (149 genes), and endocrine system (120 genes). Annotation using the CAZy database identified a total of 657 CAZyme genes, which were classified into 6 major categories (**Figure 5B**; **Supplementary Table S5**). The majority of these genes were associated with glycoside hydrolases (269 genes, 40.94%), followed by auxiliary activities (180 genes, 27.40%), and carbohydrate esterases (101 genes, 15.37%). Analysis with the PHI database enabled the annotation of 1058 genes related to host interactions, which were categorized into 7 major classes (**Figure 5C**; **Supplementary Table S6**). The predominant categories included reduced virulence (523 genes, 44.32%), unaffected pathogenicity (392 genes, 33.22%), and loss of pathogenicity (128 genes, 10.85%). Among all annotated genes, 1439 were identified as secreted proteins (**Supplementary Table S7**). The majority of these secreted proteins (1405 genes, 97.64%) lacked transmembrane domains, while a small fraction (34 genes, 2.36%) contained transmembrane domains. Additionally, 1622 transmembrane proteins were identified (**Supplementary Table S8**). Annotation using the DFVF database uncovered two virulence factors, *yidC* and *ffh*, both of which are associated with the Sec-SRP secretion system. AntiSMASH prediction identified 52 potential secondary metabolite biosynthetic gene clusters (BGCs), covering 10 major categories, including 22 type I polyketide synthase (T1PKS) clusters, 8 terpene clusters, 7 non-ribosomal peptide synthetase (NRPS) clusters, 7 NRPS-like clusters, 3 isocyanide clusters, 1 betalactone cluster, 1 terpene-precursor cluster, 1 NRP-metallophore cluster, 1 phosphonate cluster, and 1 terpene-precursor cluster (**Supplementary Table S9**).

### Genome based phylogenetic analysis of *P. nobilis* SFJ-12-R-5

**Figure 6:**
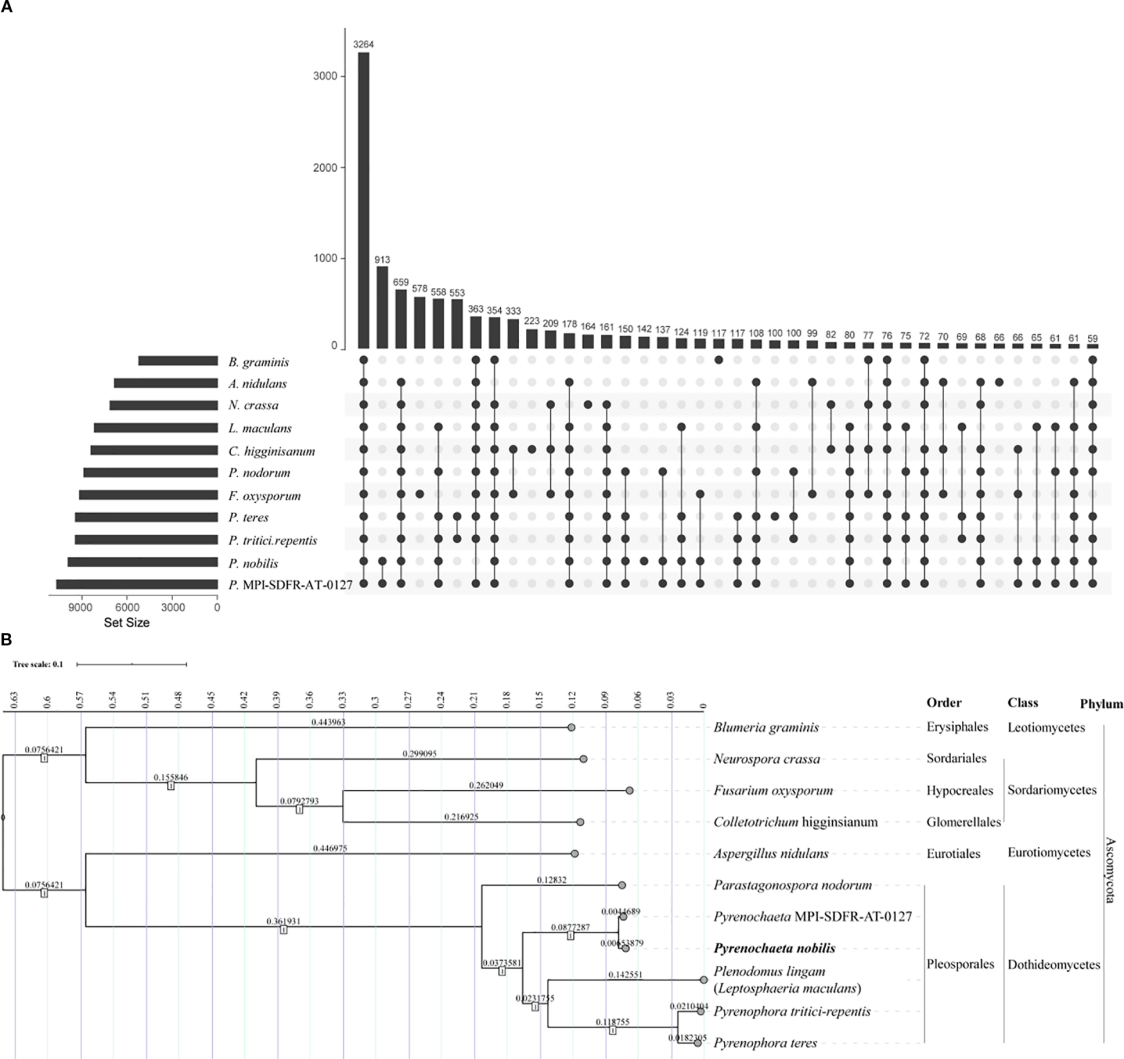
Gene family identification and whole genome based phylogenetic analysis of *P. nobilis* SFJ-12-R-5. **(A)** Upset graph showing the shared orthologous gene groups of the 11 species. **(B)** The neighbor-joining tree was constructed using super-genes formed by the tandem merging of common single - copy genes from these 11 species. The tree was generated using MEGA 11 with a bootstrap value of 1000.

To further clarify the phylogenetic relationship of *P. nobilis*, we selected the genomes of 10 reference species for in-depth comparison (**Supplementary Table S10**). By aligning the amino acid sequences of the whole coding genes, a total of 3,264 shared orthologous gene groups were identified among these 11 species (**Figure 6A**). After concatenating these genes, we used the software MEGA to construct the neighbor joining phylogenetic tree (**Figure 6B**). The results showed that all species within the order *Pleosporales* clustered together in one branch. The species most closely related to *P. nobilis* was another species within the same genus, which has not been classified at the species level, and the two species clustered together in one branch. *P. nobilis* is evolutionarily close to the genera *Leptosphaeria/Plenodomus*, followed by the genus *Parastagonospora*.

## Discussion

Greenhouse pests and diseases stand out as key challenges in Integrated Pest Management (IPM). Under favorable temperature and humidity conditions, greenhouse crops are more prone to infection by *B. cinerea*, the causal agent of gray mold. Notably, biocontrol agents exhibit effective inhibition on the proliferation of *B. cinerea*. Consequently, research focusing on these biocontrol agents is of critical importance for the prevention of gray mold in greenhouse crops. There are over one million known species of endophytic fungi in plants, making them a prominent focus of research for discovering novel and valuable bioactive compounds (De Silva et al., 2019). Due to their unique growth environments and symbiotic relationships with their host plants, endophytic fungi from medicinal plants often produce bioactive compounds similar to those found in their host. These microbial secondary metabolites can be directly developed into agricultural antibiotics to combat plant diseases and pests (Rondot and Reineke, 2018; Hummadi et al., 2021). The protective mechanisms of endophytic fungi primarily stem from their strong spatial and nutritional competitiveness. They can either release metabolites to directly inhibit plant pathogen growth or, indirectly, stimulate the host’s defense mechanisms and promote its growth.

*Astragalus* has a long-standing history of medicinal use and is one of the most commonly used bulk medicinal materials in modern Chinese medicine (Xu et al., 2024). However, research on *Astragalus* has primarily focused on its medicinal benefits. *Pyrenochaeta* spp. are commonly found in the environment and act as saprophytes in soil, plants, and wood, particularly in tropical and subtropical areas (Soltani and Hosseyni Moghaddam, 2015; Wazny et al., 2021; Behnke-Borowczyk et al., 2023). Although *Pyrenochaeta* spp. are thought to potentially cause yield losses in some crops (Lyu et al., 2020), their antagonistic potential against plant pathogens has received limited attention. In this study, we screened the antagonistic endophytic fungi *P. nobilis* strain SFJ-12-R-5 from the root of *A. membranaceus.* These findings not only underscore the significance of medicinal plants as a critical resource for screening antagonistic endophytic species but also challenges long-held perceptions, thereby opening up a new avenue for the development and application of *Pyrenochaeta* spp.

In this study, we, for the first time, systematically evaluated the antifungal activity of *P. nobilis* strain SFJ-12-R-5, both the live strain and its fermentation filtrate, against *B. cinerea* using a comprehensive suite of assay methods. In contrast to *Trichoderma*, whose biocontrol efficacy against phytopathogens primarily relies on mycoparasitism and the induction of plant systemic resistance, *P. nobilis* strain SFJ-12-R-5 was found to exert potent suppressive effects on *B. cinerea* specifically through the production of fungistatic secondary metabolites. This result was further confirmed using the Oxford Cup test. Through the inoculation of detached leaves, fruits, and whole plants, with *B. cinerea* and simultaneous application of *P. nobilis* fermentation filtrate, we observed a significant reduction in lesion areas on both leaves and fruits of leaves and fruits. These results demonstrate the reliability of *P. nobilis* strain SFJ-12-R-5 in inhibiting *B. cinerea* on tomato. It causes no harm to either leaves or fruits, confirming that *P. nobilis* is effective in controlling gray mold in tomatoes and holds promise as a biocontrol agent against *B. cinerea*. Currently, its effectiveness has only been observed in tomatoes. However, it holds promise for application in different crops, which will further broaden its scope of use.

Genomes serve as a critical foundation for investigating the functions and mechanisms of species (Rhie et al., 2021; Du et al., 2022; Xie et al., 2025). In this study, we constructed a high-quality genome of the SFJ-12-R-5 strain using short-read and long-read sequencing technologies. This will provide a robust foundation for subsequent research endeavors. Although the assembly quality of this genome is relatively high, with a contig N50 of 435.95 Kb and a BUSCO completeness of 91%, it was constrained by the technical limitations at the time the experiment was conducted. Therefore, in future studies, HiFi and Hi-C technologies can be considered to further improve the genome assembly quality (Han et al., 2025; Yang et al., 2025).

A total of 14,578 coding genes were annotated in the genome of *P. nobilis* strain SFJ-12-R-5. Through analysis using KEGG, CAZy, PHI, and DFVF databases, we gained a preliminary understanding of the species’ metabolic functions, carbohydrates metabolism, host interactions, and virulence. Ultimately, approximately 80% of the genes were functionally annotated. This annotation rate is relatively low compared to that of other widely studied species. However, with the further advancement of functional genomics and the continuous expansion of diversity in public databases, this rate is expected to increase further.

A large number of secondary metabolic gene clusters were detected in the genome of *P. nobilis* strain SFJ-12-R-5, among which T1PKS were the most abundant. T1PKS are key enzymes in secondary metabolism, primarily functioning to catalyze the biosynthesis of polyketides, which are structurally diverse and biologically active natural products (Wang et al., 2020a). This suggests that polyketide compounds may be important factors contributing to the biocontrol activity of *P. nobilis* (Senabio et al., 2023; Yan et al., 2024), warranting focused attention in subsequent studies. Although only two virulence-associated genes, *yidC* and *ffh*, were identified in this genome, both serve as core components of the Sec-SRP secretion system. As one of the pivotal pathways governing protein targeting and membrane insertion, this system plays a central role in the precise delivery of nascent proteins synthesized intracellularly to the cell membrane, or in facilitating their insertion across the membrane (Dalbey et al., 2014). Based on this functional context, we hypothesize that *yidC* and *ffh* may indirectly support the biosynthesis of secondary metabolites by ensuring the accurate localization and structural-functional integrity of synthases, such as T1PKS. In turn, this metabolic support is likely to contribute to the biocontrol capacity of the fungus.

In this study, although we successfully constructed a high-quality genome, we have not yet further elucidated its underlying mechanism. In the future, integrated analyses of multi-omics data, such as transcriptomics and metabolomics, should be performed to clarify the antagonistic mechanism of *P. nobilis* against *B. cinerea* (Shi et al., 2024; Yu et al., 2024; Zhu et al., 2025). Based on the genomic data, transcriptomic and metabolomic analyses can be conducted to further clarify gene expression patterns, identify differential metabolites, and characterize key metabolic pathways, thereby narrowing down the scope of investigation into potential inhibitory mechanisms. Building on these findings, the functions of potential key genes can be further verified through molecular techniques, such as CRISPR-based gene knockout and complementation experiments.

In conclusion, we report the isolation and genome of a novel biocontrol fungal endophyte, *P. nobilis* strain SFJ-12-R-5, which exhibits strong inhibition of the gray mold pathogen *B. cinerea*. These findings provide valuable genomic and experimental resources for future research on gray mold disease prevention and the development of sustainable biocontrol strategies. Besides, these data and analysis results further enhance our understanding of this species and lay the foundation for future research on the inhibitory mechanisms of *Pyrenochaeta* against *B. cinerea*.

## Data Availability

The data generated in this study have been deposited in the China National Center for Bioinformation database (https://ngdc.cncb.ac.cn/) under the assigned project number of PRJCA038396.
